# Overexpression of centromere protein K (CENP-K) gene in hepatocellular carcinoma promote cell proliferation by activating AKT/TP53 signal pathway

**DOI:** 10.18632/oncotarget.18172

**Published:** 2017-05-25

**Authors:** Haiyan Wang, Weilong Liu, Lei Liu, Chi Wu, Weigang Wu, Juan Zheng, Mingxia Zhang, Xinchun Chen, Boping Zhou, Zhiliang Gao, Jian Huang

**Affiliations:** ^1^ Department of Infectious Diseases, the Third Affiliated Hospital of Sun Yat-sen University, Guangzhou, Guangdong, China; ^2^ Guangdong Key Laboratory of Diagnosis and Treatment for Emerging Infectious Diseases, Shenzhen Key Laboratory of Infection and Immunity, Shenzhen Third People’s Hospital, Shenzhen, Guangdong, China; ^3^ Shenzhen People's Hospital, Second Clinical Medical College of Jinan University, Shenzhen, Guangdong, China; ^4^ GuangDong Provincial Key Laboratory of Liver Disease, the Third Affiliated Hospital of Sun Yat-sen University, Guangzhou, Guangdong, China; ^5^ Key Laboratory of Systems Biomedicine, Ministry of Education and Collaborative Innovation Center of Systems Biomedicine, Shanghai Center for Systems Biomedicine, Shanghai Jiao Tong University, Shanghai, China

**Keywords:** hepatocellular carcinoma, hepatocarcinogenesis, centromere protein K, up-regulation, methylation

## Abstract

Hepatocellular carcinoma (HCC) is one of the high-incidence malignant tumors with very poor prognosis. Identification of potential oncogenes is critical to discovering novel therapeutic targets for many cancers, including HCC. In our previous studies, using microarray technology, we conformed that CENP-K was overexpressed in HCCs. However, whether the overexpression of CENP-K contributes to hepatocarcinogenesis remains unclear. In this study, we found that CENP-K was significantly up-regulated in 60% (63 of 105) of HCC specimens at the mRNA level compared to adjacent non-cancerous liver specimens, as determined by RT-qPCR. Immunohistochemical staining confirmed similar results at the protein level. Interestingly, we found that the DNA methylation status of the CENP-K promoter was significantly reduced in HCC specimens with increased CENP-K expression. In addition, CENP-K mRNA expression level was positively correlated with the level of alpha-fetoprotein (AFP) (≥ 400 ng/ml) and tumor size (≥ 3 cm) (*p* < 0.05). CENP-K overexpression promoted proliferation and migration in SMMC7721 and Focus cells. In contrast, knock down of CENP-K significantly inhibited the growth of MHCC-LM3 and QGY7703 cells. Furthermore, we found that overexpression of CENP-K stimulated the tyrosine phosphorylation of the AKT and MDM2 proteins, but inhibited tyrosine phosphorylation of the TP53 protein. Our data suggest that the up-regulation of CENP-K, a potential oncotarget gene, may be modulated by epigenetic events and can contribute to hepatocarcinogenesis.

## INTRODUCTION

Liver cancer ranks as the fifth most prevalent malignancy worldwide, and is the third leading cause of cancer-related deaths [1]. According to American Cancer Society statistics, it is estimated that during 2012, 782,500 new liver cancer cases and 745,500 deaths occurred worldwide. Hepatocellular carcinoma (HCC) account for majoring (70% to 90%) of all primary malignant tumor that stem from in the liver [2]. Survival of HCC patients tends to be poor as a result of late diagnosis, high failure rate of chemotherapy and tumor recurrence. However, the cellular processes and mechanisms of HCC carcinogenesis remain poorly understood. Thus, identifying novel HCC-related signaling molecules elucidating and their molecular mechanisms for early diagnosis and effective molecular targeted therapies is important for improving survival rate and life quality of patients with HCC.

Increasing evidence suggests that kinetochore dysregulation or dysfunction leads to aneuploidy and promotes carcinogenesis [3]. Kinetochore is a protein structure on chromatids, which plays a crucial role in chromosome segregation during mitosis and meiosis [4]. Kinetochore contains at least 80 different proteins and many of these proteins are conserved between species including CENP (centromere protein)-A, -B,-C,-H, -K, -M, -N, and so on [5]. Increased levels of CENP-E and CENP-A expression have been reported in breast and ovarian cancers [6, 7]. Dalal lab demonstrated that excess CENP-A accumulates at noncentromeric locations in the human cancer genome, which alters the state of chromatin fiber and impacts chromosome fragility [8]. CENP-K is localized in the inner plate of kinetochore, which contributes to the effective assembly of CENP-A with other centromere components [9]. CENP-K was specifically upregulated in ovarian cancer cells and is correlated with poor patient survival [10]. However, the expression of those centromere proteins and biological functions in HCC still remain imcompletely understood.

In our previous study, we found that the expression of CENP-K was up-regulated in HBV-associated HCC specimens by using gene chip technology in HBV-related HCCs [11]. The goal of this study is to further characterize the differential expression, carcinogenic potential and mechanisms of CENP-K in HCC. We tested the mRNA level of CENP-K in 105 specimens, and assessed the roles of CENP-K contributed to HCC proliferation, colony formation, migration and tumorigenesis *in vitro* and *in vivo*. The DNA methylation status of CENP-K promoter was also evaluated.

## RESULTS

### Expression of CENP-K was frequently up-regulated in HCCs

**Figure 1:**
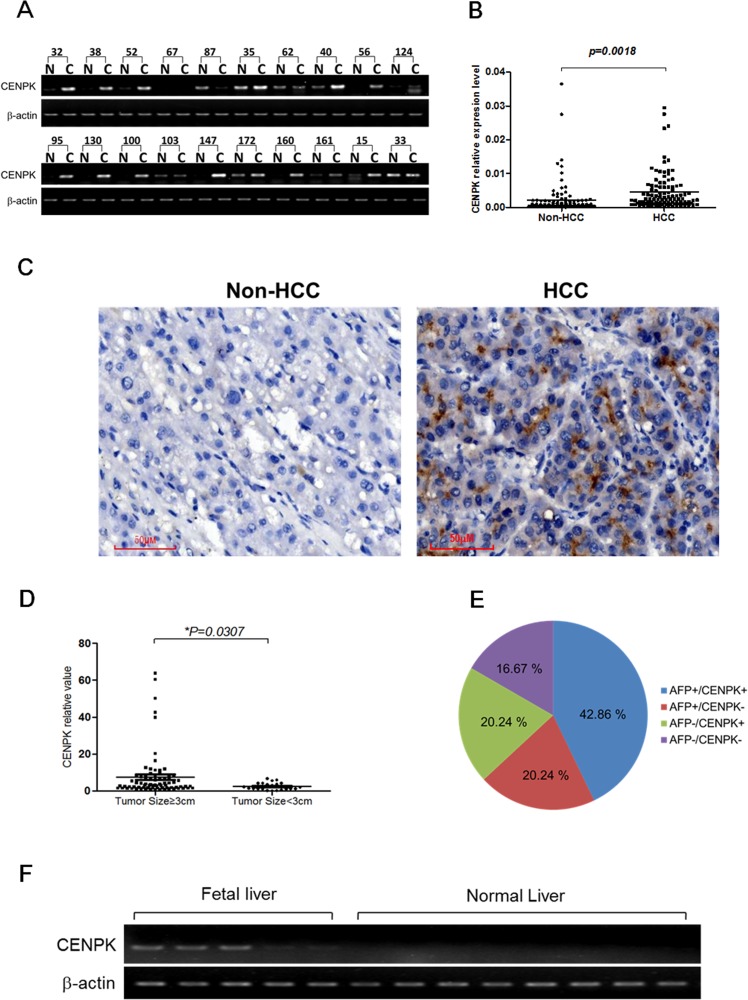
The expression pattern of CENP-K mRNA and protein in HCC specimens (**A**) Representative semi-quantative RT-PCR results of CENP-K in 20 matched HCC (C) and their corresponding adjacent non-cancerous livers (N); (**B**) CENP-K expression fold of HCC tissue versus the corresponding adjacent non-tumor tissues in 105 pairs of samples; (**C**) Representative immunohistochemical staining of a pair of HCC specimens and its corresponding non-tumor tissue, as determined using an anti-CENP-K antibody. The nuclei were counterstained with hematoxylin. (**D**) CENP-K mRNA levels comparision between tumors with the size larger or smaller than 3 cm; (**E**) The distribution of CENP-K and AFP mRNA levels in 105 HCC specimens; (**F**) Semi-quantitative RT-PCR analysis of CENP-K in 5 fetal liver tissues and 8 normal liver tissues.

To assess the mRNA levels of CENP-K in HCCs, semi-quantitative RT-PCR assay was performed. We found that CENP-K was significantly up-regulated in 14 of 20 HCC specimens (60%) as compared with those of the adjacent non-cancerous tissues (Figure 1A). Because of the limitations of the semi-quantitative RT-PCR method, the transcription of CENP-K was tested in 105 informative cases by real-time PCR to further confirm the up-regulation of this gene. The results indicated CENP-K was markedly up-regulated in 63 of the 105 (60%, more than two-fold) HCC specimens compared with adjacent non-cancerous livers (*P = 0.0018*, Figure 1B), which agrees with the result of semi-quantitative RT-PCR. Furthermore, we used immunohistochemical (IHC) staining to detect the expression of CENP-K at the protein level in HCC tissues and the adjacent non-cancerous liver tissue (Figure 1C). The results showed that CENP-K protein was significantly overexpressed in HCCs comparing with non-HCC tissues.

Interestingly, as shown in Figure 1D, the CENP-K expression was statistically correlated with tumor size (≥ 3 or < 3 cm), but not with age (≥ 65 or < 65 years), gender, HBV or lymphatic metastasis (Table 1). Moreover, overexpression of CENP-K and AFP (a-fetoprotein) did not completely overlap in the 105 HCC specimens, according to the results of real-time RT-PCR. CENP-K and AFP were both expressed in 45 of 105 HCC specimens (42.86%). Meanwhile, CENP-K was not obviously expressed in only 21 of 66 cases (31.82%) with the AFP overexpression. Notably, overexpression of CENP-K with AFP negative was 20.24% (21 of 105) of the HCC specimens (Figure 1E). These data indicate that CENP-K could be a new biomarker for HCC pathogenesis.

**Table 1:** The expression of CENPK versus clinical features

Clinicopathological parameters	Number of patients	Upregulation*	no up-regulation	***X***^2^	*P*
*Age* (years)					
* ≥ 65years*	16	11	5	0.51	> 0.05
* < 65 years*	81	48	33		
*Gender*					
* Male*	81	49	32	0.01	> 0.05
* Female*	17	11	6		
HBV					
HBV (+)	61	54	29	2.50	> 0.05
HBV (−)	6	6	8		
Tumor size					
* ≥ 3 cm*	81	48	33	0.01	< 0.05
* < 3 cm*	9	6	3		
*Lymphatic metastasis*					
* Present*	3	1	2	0.19	> 0.05
* Absent*	102	64	38		
AFP					
≥ 400 ng/ml	36	26	10	4.38	< 0.05
< 400 ng/ml	41	20	21		

*:up regulation of CENP-K was designed as ≥ 2(HCC/non-HCC).

Furthermore, we found positive expression of CENP-K in normal fetal liver tissues, compared to no expression in normal liver tissues (Figure 1F), which indicates that the expression level of CENP-K is closely related to the liver development.

### CENP-K promotes cell proliferation, cell migration and tumorigenicity

**Figure 2:**
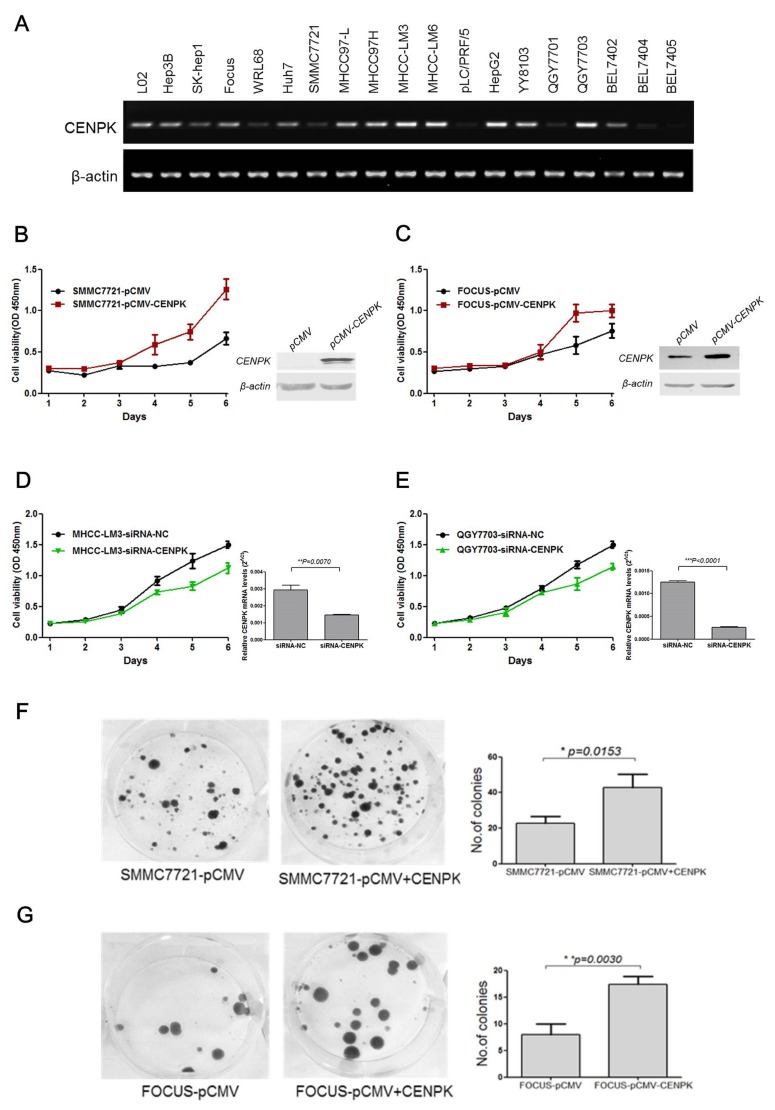
The effect of CENP-K on the growth and colony formation of HCC cells (**A**) Semi-quantitative RT-PCR analysis of CENP-K in 19 HCC cells; (**B** and **C**) CENP-K was significantly overexpressed in SMMC7721 and Focus. Blank vector pCMV was used as a negtive control. The growth curves were determined by the CCK-8 assay; (**D** and **E**) CENP-K was knockdown in MHCC-LM3 and QGY7703. SiRNA-NC was used as a negtive control. The growth curves were determined by the CCK-8 assay; (**F** and **G**) CENP-K was significantly overexpressed in SMMC7721 and Focus. Blank vector pCMV was used as a negtive control. After transfection for 24 hours, the cells were scraped and plated on dishes and cultured in G418 for 2 weeks. The representation dishes showed that CENP-K promoted the colony formation. The histogram showed that the colony formation was promoted by CENP-K, compared with the vector-only control. All the expriments were repeated at least three times and the spots represent the average values, with standard deviations (SDs) included for each mean value.

To investigate the biological function of *CENP-K* on HCC cells, we first assessed the mRNA level of *CENP-K* in various HCC cell lines and two liver-derived cell lines. Our results showed that *CENP-K* was significantly expressed in LO2, Hep3B, Huh7, MHCC97L, MHCC97H, MHCC-LM3, MHCC-LM6, HepG2, YY8103, QGY7703, and BEL7402 cell lines, whereas weak expression of this gene was found in SK-hep1, WRL68, SMMC7721, Focus, PLC/PRF/5, QGY7701, BEL7404 and BEL7405 cell lines (Figure 2A). In this study, we used SMMC7721 and Focus cell lines with low expression of CENP-K, and QGY7703 and LM3 cell lines with strong expression of CENP-K, as models to explore the function of CENP-K on HCC cells.

To evaluate whether *CENP-K* can act as an oncogene, we evaluated the effect of CENP-K overexpression or knockdown on cell proliferation, and cell migration. We transfected SMMC7721 and Focus cells with a pCMV vector containing a *CENP-K* construct, which showed relatively weak expression of this gene (Figure 2A), using an empty vector as negative control. As shown in Figure 2B and Figure 2C, CENP-K was obviously overexpressed in SMMC7721 and Focus cells. The proliferation of both cell lines was effectively increased, as compared with the negative control. On the other hand, we performed CENP-K knockdown with siRNA in MHCC-LM3 and QGY7703 cell lines which showed strong expression of this gene (Figure 2A). Compared with the cells transfected with siRNA-NC as a control, the proliferative ability of both cell lines that were transiently transfected with siRNA-CENP-K was significantly inhibited, as shown in Figure 2D and Figure 2E. Moreover, in colony formation assay, we found that the ability of SMMC7721 and Focus cells to undergo colony formation was enhanced (Figure 2F and Figure 2G).

**Figure 3:**
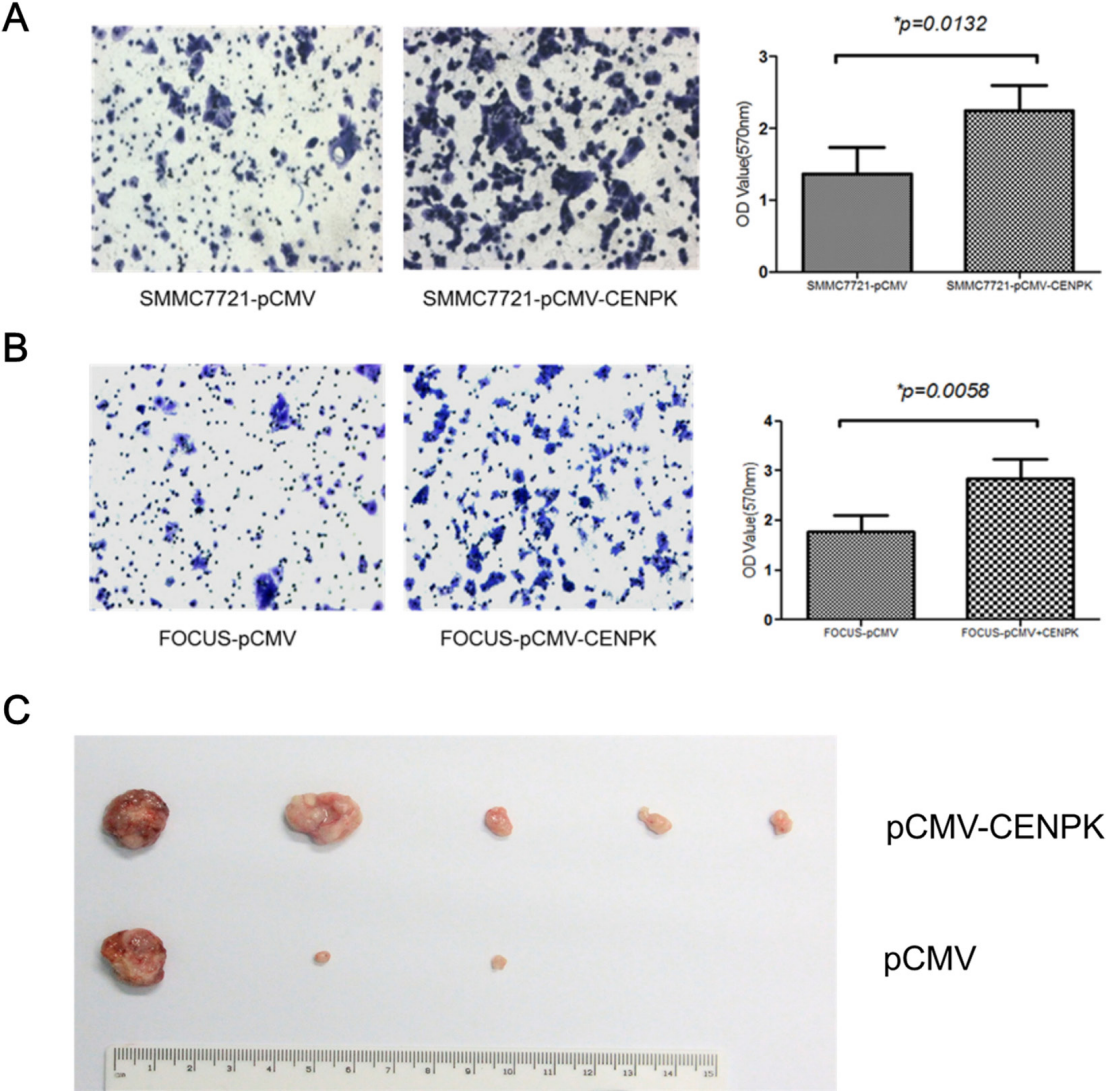
The effect of CENP-K overexpression on HCC cell migration and tumorigenicity (**A** and **B**) Blank vector pCMV was transfected into SMMC7721 (A) and Focus (B) cells as the negtive controls. The number of migrated cells is represented by the mean values per field (from at least 5 fields) from three independent experiments; (**C**) Photograph of xenografts disected from nude mice which were injected subcetaneously.

We also performed the transwell assay to evaluate the effect of CENP-K on the ability of cell migration. SMMC7721 and Focus cells were cultured respectively in the upper chambers of a transwell, and 24 hours later the cells that had penetrated the membrane were collected and the concentrations were determined at OD_570nm_. For SMMC7721 cells transfected with CENP-K or an empty pCMV vector as a control, the values at OD_570nm_ were 2.4 and 1.3, respectively (Figure 3A, *p* = 0.0132). For Focus cells, the values were 3.0 and 1.9, respectively (Figure 3B, *p* = 0.0058). These results indicated that CENP-K showed significant promotion of cell migration. Subsequently, to detect the ability of tumorigenicity *in vivo*, we injected SMMC-7721 cells (1 × 10^6^ cells/mice) from each of these stable subclones subcutaneously into ten athymic mice to assess tumorigenicity. By six weeks, two larger tumors were detected in all the five mice injected with SMMC7721 cells overexoressing CENP-K protein. In contrast, only one larger tumor and two small tumors were formed in three of the five mice injected with the vector control cells (Figure 3C). Taken together, these results suggest that CENP-K plays an important role in promoting cell growth, migration and tumorigenicity.

### DNA methylation status of CENP-K promoter is reduced in HCC tissues

**Figure 4:**
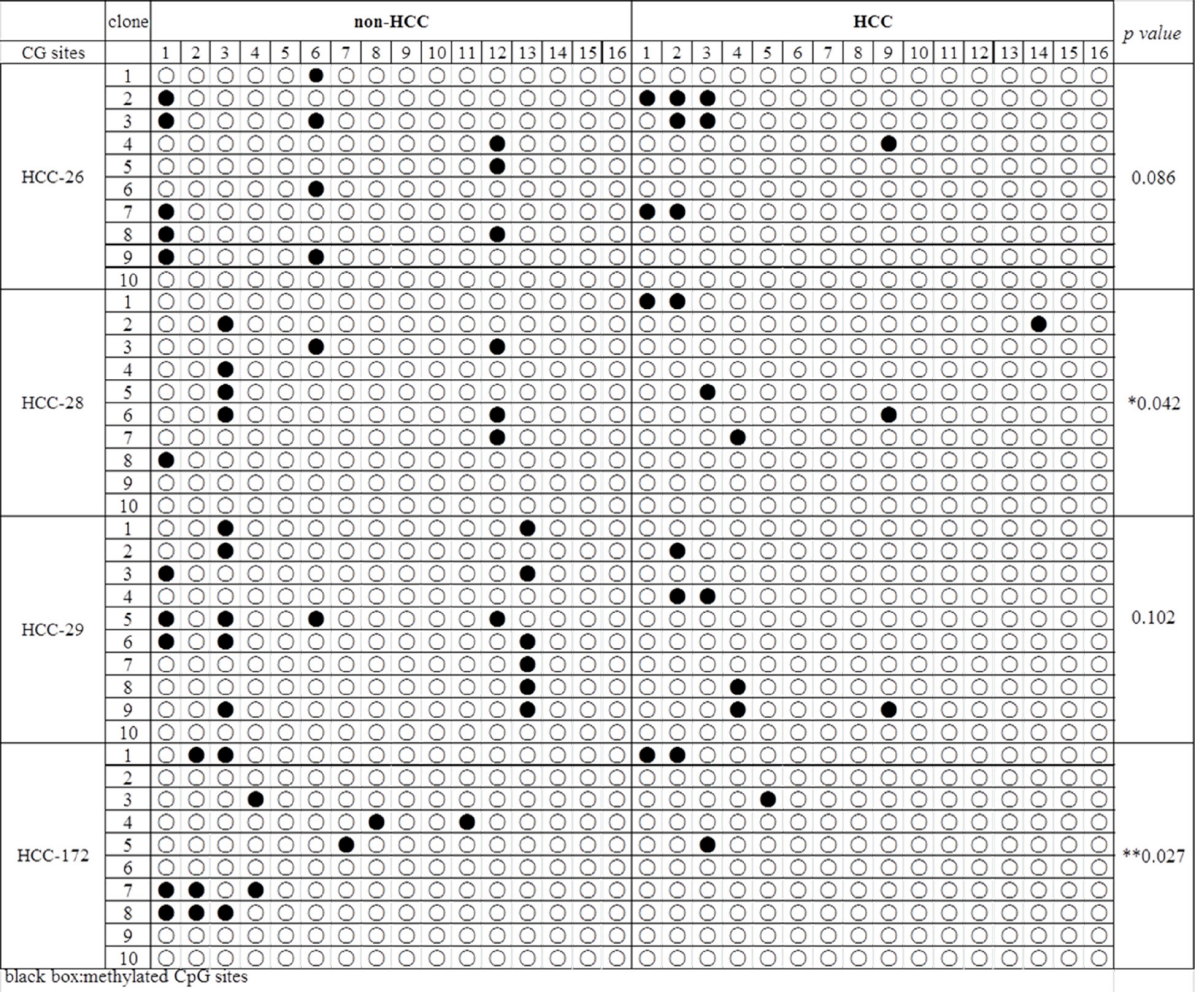
The DNA methylation status of the CENP-K promoter in HCC The results from the bisulfite sequencing analysis of *CENP-K* promoter CpG methylation in four pairs of HCC and non-HCC specimens. Each box stands for a CpG dinucleotide within the CpG island in the promoter. Black spot suggests methylation cytosine while blank box indicates unmethylated cytosine.

DNA methylation is a process that involves methyl groups being added to cytosine and adenine, catalyzed by DNA methyltransferases. DNA methylation can repress gene transcription when it occurs in a promoter. In order to determine the dysregulation mechanisms of CENP-K, we characterized the methylation status of the CENP-K promoter in four pairs of HCC and non-HCC specimens through bisulfite DNA sequencing. The sequencing results showed that CENP-K promoter methylation was obviously reduced in those four HCC specimens, compared with adjacent non-cancerous liver tissues (Figure 4). This result indicates that upregulation of CENP-K in HCC is associated with promoter methylation levels.

### CENP-K promotes focus cell migration via activation of the AKT/TP53 pathway

**Figure 5:**
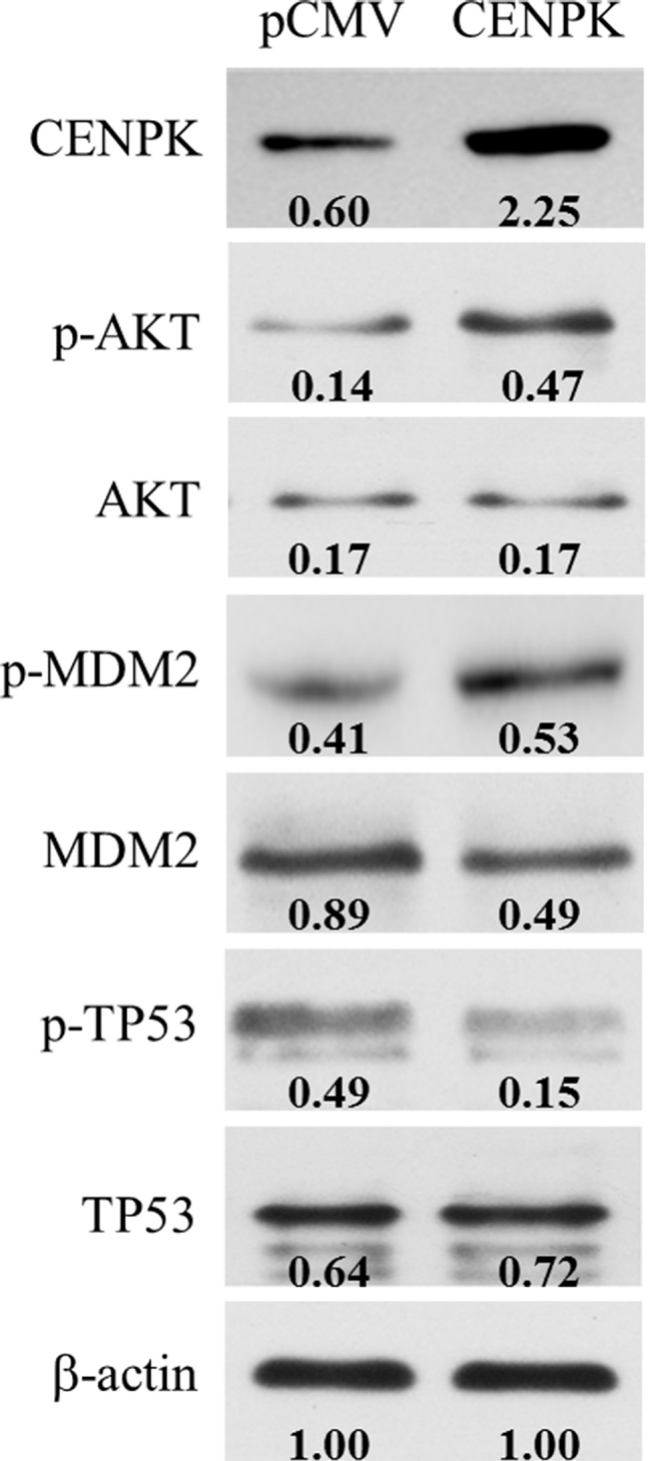
CENP-K modulates the phosphorylation of the AKT/TP53 signaling pathway The overexpression of CENP-K enhanced the phosphorylation of AKT and MDM2, and inhibited the phosphorylation of TP53 in Focus cell line. β-actin was used as a loading control. Quantification of phosphorylation and non-phosphorylation levels were indicated by the numbers which were performed by normalizing the concentrations to the β-actin control.

To further investigate the possible cellular signaling pathway involved in CENP-K-promoted cell proliferation and migration, we performed Western blotting to identify the candidate downstream genes involved in several intracellular signaling pathways, such as PI3K/AKT, FAK/AKT and p38/MKK3/6, which are considered critical pathways that contributes the cell proliferation and migration. After transfecting the CENP-K gene into Focus cells which has weakly expression of CENP-K gene (Figure 2A), we found that CENP-K protein was successfully over-expressed in Focus cells (Figure 5). Then semi-quantitative analysis assay was performed to detect the quantitative changes of total proteins and phosphorylated proteins involved in the above pathways in Focus cells with CENP-K compared with that in Focus without CENP-K. The results showed that the phosphorylation levels of AKT and MDM2 were significantly increased in Focus cells overexpressing CENP-K compared with control cells, whereas the phosphorylation of TP53 was markedly suppressed (Figure 5). The current results suggested CENP-K promotes cell proliferation and migration via an AKT/TP53-dependent mechanism.

## DISCUSSION

HCC remains the most common type of liver malignancy in less developed countries, with China alone accounting for about 50% of the total number of new liver cancer cases and deaths [2, 12]. Its development involves a large number of molecules [13, 14] and multiple steps, such as activation of oncogenes, inactivation of tumor suppressor genes, epigenetic alternations, DNA repair and chromosome segregation [15]. Kinetochores, large protein complexes assembled on the centromeric region of the chromosomes, facilitate proper chromosome segregation during cell division and proliferation [5, 16]. Large body of aberration of kinetochores expression in tumorigenesis has been observed in various cancers [10, 17, 18], including HCC. For example, a previous study showed that CENP-H was overexpressed in HCC, and that upregulation of CENP-H was an independent prognostic indicator [19]. Another study revealed that CENP-E expression was reduced in human HCC tissue, while low CENP-E expression resulted in aneuploidy in LO2 cells [20].

CENP-K is a component of kinetochores, with its gene located on chromosome 5 in humans [21]. CENP-K and CENP-H may form coiled-coils in the kinetochores [22]. CENP-H/-I/-K complex and KNL1 appear to work coordinately to target several other CENPs to kinetochores, because co-depletion of CENP-K and KNL1 abrogates centromeric localization of Hec1/Ndc80 complex [23]. CENP-H/I/K is also required for the incorporation of CENP-A into the kinetochores [9]. These findings indicate that CENP-H and CENP-K might play an essential role in kinetochore assembly and function throughout the cell cycle. To our knowledge, there is no report about CENP-K contributing to the initiation and/or progression of HCC, or the potential clinical implications of CENP-K expression in HCC patients. The present study confirms that CENP-K was highly expressed in HCC specimens compared with the adjacent liver tissues, and that the CENP-K expression was statistically correlated with tumor size. Moreover, overexpression of CENP-K and AFP levels did not completely overlap in the HCC specimens. We found that the expression level of CENP-K was upregulated in 20.24% (21 of 105) HCC cases with AFP negative expression. AFP have been widely used as a biomarker for the diagnosis and surveillance of HCC. However, the sensitivity and specificity of AFP for HCC surveillance have some limitation because the AFP levels may be normal in up to 40% of HCC patients, particularly during the early stage of the disease [24]. Thus, based on evidence in our study, detecting AFP and CENP-K simultaneously could improve diagnostic accuracy. Furthermore, the expression level of CENP-K varied in different HCC cell lines, and was high in fetal liver while there was very low or even no expression in the normal liver tissues. Therefore, it may be speculated that CENP-K plays an important role in maintaining the malignant phenotype of HCC cells, and it might be a gene that is closely related to HCC.

Next, we validated the function of CENP-K in different HCC cell lines by applying loss-of-function or gain-of-function approaches. In MHCC-LM3 and QGY-7703 cell lines, knockdown of CENP-K with siRNAs could inhibit cell proliferation. On the other hand, in SMMC-7721 and Focus cell lines, overexpression of CENP-K promoted cell proliferation, colony formation and cell migration *in vitro*. We injected two groups of transfected SMMC-7721 cells (1 × 10^6^ cells/mice) subcutaneously into five athymic mice, after six weeks the result showed that the tumorigenesis ability of CENP-K remarkably increased compared to the vector control *in vivo*. This is the first report to demonstrate the functional significance of CENP-K overexpression in HCC, and our findings implicate CENP-K as an oncogene that promotes malignant HCC progression.

Finally, one regulatory mechanism governing CENP-K expression was investigated, by characterizing the methylation status of the CENP-K promoter region in four pairs of HCC and non-HCC specimens with the designed primers. Aberrant methylation of CpG-rich islands in or near gene promoter region has been associated with transcriptional inactivation of oncogenes or tumor suppressors in human cancers including HCC [25–27]. The HCV core protein has been reported to significantly down-regulate the expression and the function of pRb2/p130 protein by inducing pRb2/p130’s promoter hyper-methylation accompanied with the up-regulation of DNMT1 and DNMT3b expression [28]. Recently, methylation of the GATA5 promoter was observed to be associated with the age of patients exhibiting HCC, and restoration of GATA5 expression could inhibit colony formation and induced apoptosis of HCC cells *in vitro* [29, 30]. Our results showed that CENP-K promoter methylation was markedly reduced in those four HCC specimens compared with the adjacent non-cancerous liver tissues, suggesting that the expression of CENP-K was regulated by the CpG-rich island methylation in the promoter. In addition, some published studies suggest the diagnosis and prognosis value of gene methylation in HCC [31]. RASSF10 is frequently hypermethylated and down-regulated in HCC, which is correlated with increased tumor recurrence and reduced survival in HCC patients. Therefore, RASSF10 can potentially serve as a useful biomarker predictive of HCC patient prognosis [32]. Meanwhile, we provided evidence that CENP-K promote cell proliferation via regulation of AKT/TP53 signaling pathway. The AKT/TP53 signaling pathway is a critical pathway in cell proliferation and tumor growth. In this study, our data showed that the phosphorylation of TP53 was suppressed in Focus cells in conjunction with overexpressed CENP-K, whereas the phosphorylation of AKT and MDM2 was enhanced (Figure 5). These results support the notion that CENP-K plays important roles in HCC carcinogenesis and was upregulated by promoter undermethylation, and that overexpression of CENP-K may promote HCC proliferation by activating AKT/TP53 signaling pathway. However, other regulatory molecular mechanisms remain unclear and need to be further investigated.

Centromeric chromatin is consisted of centromeres and kinetochores. It is well known that the misregulation of centromeres contributes to chromosomes mis-segregation, which is closely related to cancer and abortion. Notably, the overexpression of many centromere genes show strong relevant to cancer malignant phonotype, but the mechanisms are still incompletely understood [33]. In this study, for the first time we clearly demonstrated the CENP-K expression situation in large clinical HCC specimens, fetal and normal liver tissues, and various HCC cell lines. Moreover, our work highlights a crucial role for CENP-K acting as an oncogene in the development and progression of HCC, and mechanistic analysis reveals a regulatory pathway of CENP-K overexpression by reducing promoter methylation. Based on these results, CENP-K has been shown to be a potential therapeutic target and diagnostic indicator for HCC.

## MATERIALS AND METHODS

### Patients, tissues specimens and cell lines

The 105 pairs of HCC tissues and their adjacent non-HCC tissues used in this research were obtained from the livers of HCC patients who underwent surgical tumor resections at the Third People’s Hospital between November 2001 and April 2010. The clinicopathological characteristics of these 105 patients, including the age, gender, hepatitis B surface antigen (HBsAg), alphafetoprotein (AFP) level, the size of tumors, and lymphatic metastasis were collected, and the results are summerized in Table 1. Meanwhile, eight normal tissue samples were taken from the surrounding tissues of patients with hepatic hemangioma, and five fetal tissue samples were obtained from aborted fetuses in the obstetrics department of the hospital. All these samples were frozen in liquid nitrogen and stored at −80°C once they were got. Liver cell lines (including LO2 and WRL68) and HCC cell lines (including Hep3B, SK-hepI, Focus, Huh7, SMMC7721, MHCC97L, MHCC97H, MHCC-LM3, MHCC-LM6, PLC, HepG2, YY8103, QGY7703, BEL7402, BEL7404, BEL7405) were from Shanghai (Shanghai Center for Systems Biomedicine, Shanghai Jiao Tong University) and cultured in our lab.

### RNA extraction and cDNA synthesis

Total RNA was isolated from frozen tissue samples or HCC cell lines using the Rneasy Mini Kit (Qiagen). First-strand cDNA was synthesized using the PrimeScript 1^st^ strand cDNA Synthesis Kit (TAKARA) according to the manufacturer’s instructions.

### Semi-quantitative RT-PCR and quantitative real-time PCR

Semi-quantitative RT-PCR was performed using the cDNA as the template and the following primers for CENP-K: 5′-GTTTGTGACGCTGTGATGGTCT-3′ (forward) and 5′-ACGCTTGAGGATGCAAGATGT-3′ (reverse). The length of the amplified fragment was 121 bp. The primer sequences for the internal reference gene b-actin were 5′-GGACTTCGAGCAAGAGATGG-3′ (forward) and 5′-AGCACTGTGTTGGCGTACAG-3′ (reverse). The length of the b-actin was 234 bp.

### Immunohistochemistry (IHC)

Freshly dissected tissue was fixed with formalin, and embedded in a paraffin block. Section the paraffin, embedded tissue block at 3 µm thickness on a mocrotome and float in a 45°C water bath containing distilled water. Transfer the sections onto glass slides and allow the slides to dry overnight. The slides were then deparaffinized in xylene and rehydrated in a graded ethanol series. Antigen retrieval was performed by pressure cooking for 2.5 min in ethylenediamine tetraacetic acid (EDTA) buffer (pH = 8.0). Incubate the sections in 3% H_2_O_2_ solution at room temperature for 10 min to block endogenous peroxidase activity. Allow the slides to cool to room temparature and washed them in phosphate-buffered saline (PBS). Subsequently, the slides were incubated with a rabbit anti-human CENP-K antibody (lot^#^:LS-B9423, LifeSpan BioSciences, 1:200 dilution) at 37°C for 1 h, washed with PBS, incubated with an anti-rabbit antibody for 1 h at room temperature and then washed with PBS again. Stained the slides with 3,3-diaminobezidine tetrahydrochloride (DAB). Finally, the sections were counterstained with Mayer’s hematoxylin, dehydrated, and mounted.

### Western blotting

Total cellular protein was extracted using lysis buffer (Beyotim Biotechnology). Protein concentration was measured using Nanodrop. Equal amount of protein samples were separated by 12% SDS-PAGE and transferred to PVDF membranes (BioRad). The membranes were blocked with 5% BSA in PBS buffer and incubated with primary antibody overnight at 4°C. β-actin was used as internal positive control. After applying a secondary antibody followed by horserdish peroxidase conjugated, immunodetection was performed with enhanced chemiluminescence, detected on X-ray films (Fuji films). A semi-quantitative analysis assay of the results was performed through the software Gel-Pro analyzer (Ver. 4.0), with the bands of b-actin as baselines.

### CENP-K overexpression and RNA interference

Two siRNAs against *CENP-K* were designed and chemically synthesized by GenePharma Con., Ltd (Shanghai). These two siRNAs target different coding regions of *CENP-K* and the sequences are as follows: siRNA1 5′-CUGCCUGAUAGAAGUGUUATT-3′ (antisense strand 5′-UAACACUUCUAUCAGGCAGTT-3′), and siRNA2 5′-GCUCAGCUAUCAUUGUUAATT-3′ (antisense strand 5′-UUAACAAUGAUAGCUGAGCTT-3′). The sequence used for the negative control was 5′-UUCUCCGAACGUGUCACGUTT-3′ (antisense strand 5′-ACGUGACACGUUCGGAGAATT-3′) which was supplied and synthesized by GenePharma Con., Ltd.

### Cell growth assay, colony formation and cell migration assay

All of the siRNAs were transfected into different HCC cell lines, and cell growth was monitored. For siRNA transfection, 3 × 10^3^ HCC cells per well were seeded in 96-well plates. When the cells reached 30%–50% confluence, they were transfected with the synthetic siRNAs at a final concentration of 50 nM using Lipofectamine 2000 Transfection Reagent (Invitrogen) accordng to the manufacturer’s instructions. Briefly, 10 µl CCK-8 solution was added to each well of the plate, and the plate was incubated at 37°C for 1 hour. The absorbance was measured at 450 nm to assess cell viability. All experiments were independently repeated at least three times.

SMMC7721 and Focus cells in 100 mm dishes were transfected with pCMV-CENP-K or pCMV vector as control in Lipofectamine 2000 (Invitrogen) for 24 hours, respectively, and were subsequently selected on G418 (0.6–1.0 mg/ml) (Invitrogen) for 3–4 weeks. Subsquently, the cells were fixed and stained, and the colonies were counted. Cell invasion assays were performed using 24-well Transwells (8 µm pore size; BD Biosciences) that were coated. HCC cells were starved overnight n serum-free medium, trypsinized and washed three times in DMEM containing 1% FBS. A total of 1 × 10^5^ cells were then resuspended in 500 µl DMEM containing 1% FBS and added to the upper chamber, while 750 µl DMEM containing 10% FBS and 10 µg/ml fibronectin (BD Biosciences, San Jose, CA, USA) was placed in the lower chamber. For the control, medium containing 1% FBS was added to the lower chember. After 48 hours of incubation, the the cells remaining in the upper chamber were removed by cotton swabs. The cells on the lower surface of the membrane were fixed in 4% paraformaldehyde and stained with 0.5% crystal violet. The cells in at least six random microscopic fields (magnification, × 100) were counted and photographed. All experiments for observing colony formation and cell invasion were independently repeated at least three times.

### Bisulfite DNA sequencing

Genomic DNA was extracted from the tissue samples using the Universal Gen DNA Kit (Cwbiotech, CW2298) according to the manufacturer’s instructions. Bisulfide treatment of the genomic DNA was performed using the EpiTect Bisulfite Kit (Qiagen) according to the manufacturer’s instructions. For bisulfite DNA sequencing, a pair of primers which are specific to the *CENP-K* gene promoter were disigned as follows, 5′-TATTTTGGTTAATATGGTGAAATTT-3′ (forward) and 5′-CATTTTAAATATAATCTAAACTCAAATC-3′ (reverse), then the CpG site was amplifided using these primers. The PCR products were purified and subcloned into the pMD18-T vector (TaKaRa). Random colonies were selected for sequencing.

### Statistical analysis

All data in this paper were displayed as mean ± SD. The statistical tool we used was GraphPad Prism 5. Statistical differences between two groups were performed by Students *t*-test or chi-square test. *P* value < 0.05 were considered indications of statistical difference.
